# Evaluation of Gingival Sulcus Width Gain After Nd: YAG Laser and Astringent Retraction Paste Using Intraoral and Laboratory STL Analysis: A Pilot Split-Mouth Study

**DOI:** 10.3390/jcm15062459

**Published:** 2026-03-23

**Authors:** Edwin Sever Bechir, Andrei-Mario Bădărău-Șuster, Mircea Suciu, Anca-Georgiana Zamfir, Zsuzsanna Bardocz-Veres, Farah Bechir

**Affiliations:** 1 Department of Oral Rehabilitation and Occlusology, Faculty of Dental Medicine, George Emil Palade University of Medicine, Pharmacy, Science and Technology of Targu Mures, 38 Gh. Marinescu Str., 540142 Targu Mures, Romania; edwin.bechir@umfst.ro (E.S.B.); mircea.suciu@umfst.ro (M.S.); zsuzsanna.bardocz-veres@umfst.ro (Z.B.-V.); 2 Doctoral School of Medicine and Pharmacy, George Emil Palade University of Medicine, Pharmacy, Science and Technology of Targu Mures, 38 Gh. Marinescu Str., 540142 Targu Mures, Romania; 3 Faculty of Dental Medicine, “George Emil Palade” University of Medicine, Pharmacy, Science and Technology of Targu Mures, 38 Gh. Marinescu Str., 540142 Targu Mures, Romania; zamfiranca21@gmail.com; 4 Department of Preventive and Community Dentistry, Faculty of Dental Medicine, “George Emil Palade” University of Medicine, Pharmacy, Science and Technology of Targu Mures, 38 Gh. Marinescu Str., 540142 Targu Mures, Romania; farah.bechir@umfst.ro

**Keywords:** gingival displacement, Nd: YAG laser, astringent retraction paste, intraoral scanning, laboratory scanning, STL analysis, digital dentistry

## Abstract

**Background/Objectives**: Advancements in digital dentistry have led to new approaches for soft tissue management aimed at improving impression accuracy. This pilot split-mouth study included a single 39-year-old male patient with 19 abutment teeth (114 measurement points). Sulcus width gain was measured at six standardized points per abutment tooth (mesio-buccal, centro-buccal, disto-buccal, disto-oral, centro-oral, mesio-oral) using Exocad software. **Methods**: Nineteen abutment teeth (114 measurement sections) from one patient were included in a randomized split-mouth design. Gingival displacement was performed either with a Nd: YAG laser or astringent retraction paste. Sulcus width gain was measured at six standardized points per abutment using Exocad software version 3.1 on superimposed STL files obtained by intraoral (IOS) and laboratory (LABSCAN) scanners. Statistical analysis was conducted in JASP (α = 0.05). **Results:** Both gingival displacement methods achieved sufficient sulcus widening above the clinical threshold of 0.20 mm. Mean gains ranged from 0.270 mm (LASER, IOS) to 0.378 mm (PASTE, LABSCAN). Intergroup comparisons revealed no statistically significant differences between the two gingival displacement methods (*p* > 0.05), whereas a significant difference was found between scanning modalities (*p* < 0.001), with higher values recorded for the laboratory scanner. The results should be interpreted cautiously due to the pilot design and limited sample size. **Conclusions:** Both techniques proved clinically effective for soft tissue displacement, with the laboratory scanner yielding higher sulcus width measurements. As a preliminary investigation, these findings should be interpreted cautiously due to the pilot nature of the study and the inclusion of a single participant.

## 1. Introduction

To achieve a successful, well-fitting, fixed dental prosthesis, it is imperative to attain a precise impression that can provide the dental technician with the most accurate visual information of the preparation margins.

Even if the supra-gingival margins of restoration are the most beneficial regarding good periodontal health maintenance, many clinicians tend to opt for juxta-gingivally or sub-gingivally placed finishing lines to ensure the best possible aesthetics of the final prosthesis [1].

Gingival displacement can be performed through mechanical means like retraction cords, chemical systems such as astringent pastes, or surgical methods including laser troughing. Although retraction cords are still considered the gold standard, newer options like paste-based displacement and laser-assisted troughing have been developed to streamline clinical procedures and enhance patient comfort [2].

Therefore, the gingival sulcus must be sufficiently deflected, which means, following the literature findings, that a gingival displacement (gingival retraction) of a minimum 0.20 millimetres is needed [2,3,4]. This retraction allows the impression material to flow properly, capturing critical areas of the tooth that would otherwise be obscured by the gingival margin [3]. The goal of this process is to provide enough space for the impression material, both vertically and horizontally, and eventually result in an impression without voids and tears, which precisely represents the margins of the restoration [4].

Achieving an adequate gingival sulcus width before impression-taking is critical for improving the visibility of preparation margins in both conventional and digital workflows. This enhanced clarity allows for more precise margin placement and accurate finish line detection, leading to restorations with superior marginal adaptation. A well-defined sulcus facilitates the fabrication of restorations with accurately contoured margins, thereby reducing the risk of overhangs or under-contoured areas, minimizing microleakage and the incidence of secondary caries, and ultimately enhancing the long-term success and durability of the final restoration.

Furthermore, an accurate impression is essential to ensure that the prosthetic restoration does not invade the biological width, preserving periodontal health and preventing inflammatory complications. Proper marginal adaptation and respect for the biological space are crucial for maintaining tissue stability and ensuring the long-term success of the restoration [5].

For both conventional and digital impression techniques, there are two key elements in achieving precise reproduction of the finish area: the first one is having a dry and clean field and the second one is having the gingival tissue pushed away to record this area, both goals being attained while maintaining the integrity of the tissue. The management of the soft tissue or gingival displacement can be obtained using numerous reversible gingival retraction methods, such as mechanical and mechano-chemical systems, surgical techniques, and laser troughing [6].

This study emphasizes the necessity of staying aligned with advancements in dental technology, particularly in soft tissue management and impression techniques, as digital innovations continue to reshape dental practices. By evaluating and comparing two emerging methods, the research aims to assess their effectiveness in clinical applications. Given the growing prevalence of digital workflows in dentistry, these insights are essential for optimizing clinical outcomes and improving procedural efficiency.

This study compares the use of a Nd: YAG laser and retraction paste as alternatives to traditional retraction cords, as we aimed to conduct an original study by selecting two novel techniques. Retraction cords, while commonly used, can be challenging to handle and time-consuming [7,8,9]. By evaluating these alternative approaches, the research seeks to determine their effectiveness in achieving gingival retraction while providing a more practical and efficient solution for clinicians. The diode laser has already been used for gingival troughing, but it has some negative effects following its use, such as pain and postoperative inflammation. Information on the use of the Nd: YAG laser for this purpose is scarce, but its use has been associated with faster healing, less inflammation and reduced bleeding [10].

The primary objective of this study was to evaluate and compare the immediate sulcus width gain obtained after gingival displacement using Nd: YAG laser troughing and astringent retraction paste. Measurements were performed using digital STL analysis obtained through intraoral and laboratory scanning.

The null hypothesis stated that there would be no significant differences between the two gingival displacement methods or between the two scanning techniques regarding the measured sulcus width gain.

## 2. Materials and Methods

### 2.1. Participants

This preliminary pilot study followed a randomized split-mouth design conducted on a single, non-smoking, 39-year-old male patient. A total of 19 abutment teeth (yielding 114 measurement sections) were prepared for fixed prosthodontic rehabilitation. The patient presented with a thick gingival phenotype and optimal periodontal health. The study was carried out based on the agreement dated 6 June 2023 (number 2363) of the Research Ethics Committee of the “George Emil Palade” University of Medicine, Pharmacy, Science and Technology in Târgu Mureș, following the evaluation of the submitted documents that were assessed for compliance with ethical standards and approval of the study. This research adhered to the ethical principles outlined in the Declaration of Helsinki and followed good clinical practice guidelines. The participant was recruited from the dental clinic of the institution, following approval from the Faculty of Dental Medicine at the “George Emil Palade” University of Medicine, Pharmacy, Sciences and Technology in Târgu Mureș, by the Institutional Agreement. The participant received clear information about the study protocol and was requested to provide informed consent before participation.

### 2.2. Clinical Inclusion and Exclusion Criteria

The inclusion was based on the following specific parameters:

- Periodontal Health: Absence of periodontal pockets, with probing pocket depth (PPD) < 3 mm at all sites.
- General Health: No associated general pathologies (e.g., diabetes mellitus, metabolic disorders, or immunocompromised status).
- Medication: No history of chronic medication that could influence gingival healing or inflammatory response.

### 2.3. Trial Design

This is a randomized „split mouth design” study that compares the differences in post-displacement sulcus width changes after using two different gingival deflection techniques. The 19 abutment teeth were assigned to either the Nd: YAG laser group or the astringent paste group using a simple randomization procedure. To ensure allocation concealment, the method for each tooth was determined by drawing lots from sealed envelopes. The order of gingival displacement procedures was randomized at the quadrant level using sealed envelopes. This ensured that the allocation of the laser or paste technique was not influenced by tooth position or operator preference.

This investigation was designed as a pilot study aimed primarily at testing the feasibility and methodological reliability of the proposed clinical and digital workflow. Therefore, no formal sample size calculation was performed due to the exploratory nature of the study.

To avoid selection bias and ensure clinical comparability, the allocation was balanced by dental arch. Specifically, the randomization was designed so that both gingival troughing with laser and displacement with astringent paste were performed on each arch (maxillary and mandibular). This split-mouth approach ensured that each arch received both treatments, thereby minimizing the influence of anatomical and physiological differences between the upper and lower jaws on the study outcomes. Thereby, for the first group (LASER), which was composed of maxillary left quadrant and mandibular right quadrant, gingival troughing was performed using the Nd: YAG Laser LightWalker^®^ AT S Fidelis (Fotona^®^, Ljubljana, Slovenia), while for the second group (PASTE), composed of maxillary right quadrant and the mandibular left quadrant, the deflection of the gingiva was achieved by using the astringent retraction paste from 3M^TM^ ESPE^TM^.

### 2.4. Stage and Data Obtaining

Firstly a careful clinical examination was conducted, as well as an OPG investigation. A medical history was taken that included questions about the patient’s name, sex, age, smoking habits, and the presence or absence of acute or chronic illnesses. The procedures were carried out by a single clinician with over 12 years of experience, strictly adhering to the manufacturer’s guidelines for both the laser and retraction paste. This approach ensured a calibrated application, maintaining consistency and eliminating any potential discrepancies in the technique. The periodontal health status was analyzed by recording in a periodontal chart, which included data about probing pocket depth (PPD), gingival retraction (GR), plaque index (PI), bleeding on probing index (BOP), and the mobility of the teeth. The patient was informed about the criteria of the study, and his informed consent was obtained. The oral rehabilitation was planned through fixed prosthetic restorations, and the abutment teeth were prepared during the initial clinical session using the biologically oriented preparation technique (BOPT). Afterwards, the teeth were protected with the help of provisional immediate crowns, which were obtained chairside through the Scutan method, using ProviCrown Max (Schulzer, Germany), a self-polymerizing material based on multifunctional methacrylic esters that is methyl methacrylate-free and used for provisional crowns and bridges. The provisionals were then cemented with Temp-Bond^TM^ NE (Kerr Corporation, Orange, CA, USA), a non-eugenol temporary cement for one week.

**Figure 1:**
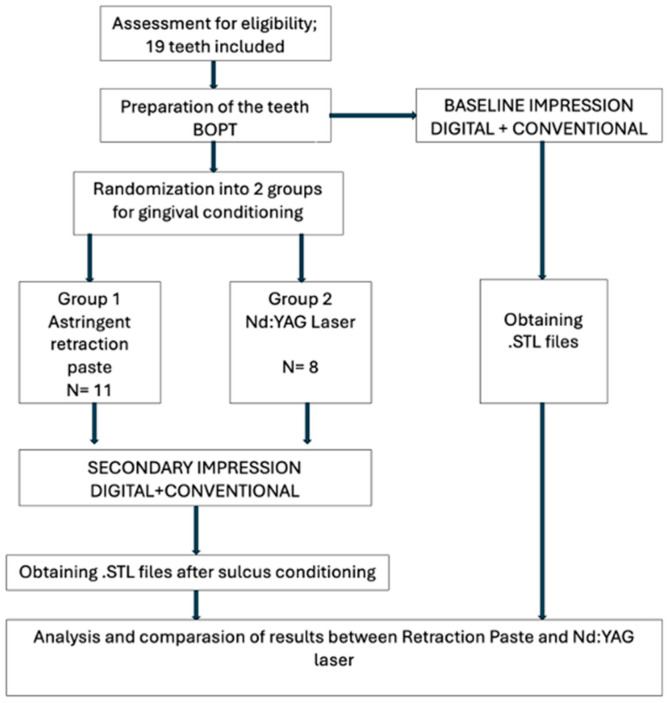
Schematic diagram of the study protocol.

A duration of 7 days was allowed for complete soft tissue stabilization before the final impression session, the patient came back to the clinic for the second visit, and the provisional crowns were gently removed without breaking them to take an impression. Two baseline impressions were taken in two different ways: one conventionally, using a polyether impression material—Impregum^TM^ Penta^TM^ Soft (3M ESPE, Seefeld, Germany), applied on a standard plastic impression tray—and a digital impression with the Medit i500 (Medit Corp., Seoul, Republic of Korea) intraoral scanner. This impression of the pre-displaced gingival tissue was very important for precisely determining the width changes obtained by the two methods of gingival deflection, permitting a direct comparison with the post-displacement values and enhancing the scientific validity of the study. Baseline STL recordings were obtained immediately before gingival displacement during the same clinical visit, ensuring that the measurements represented short-term sulcus width gain rather than tissue remodeling. The diagram of the study protocol can be visualized in Figure 1.

The intraoral scanner Medit i500 has a reported accuracy of approximately 21.0 µm ± 1.48 μm according to manufacturer specifications.

The two methods of gingival displacement that were randomly applied are presented below:

- Mechano-chemical method: 3M^TM^Astringent Retraction paste (3M ESPE, Seefeld, Bayern, Germany)
- Surgical method: Nd: YAG laser with a 1064 nm wavelength (LightWalker^®^ AT S Fidelis, Fotona^®^, Ljubljana, Slovenia)

Once the impression had been taken, the gingival trough phase followed, using the Nd: YAG laser on the first group of teeth and the astringent paste on the second group. The laser settings used for this procedure were the following: Laser Type: Nd: YAG; wavelength: 1064 nm; Mode: Short Pulse; Power: 2W and Frequency: 20 Hz. During this procedure, both the medical staff and the patient wore laser safety glasses. To activate the tip of the 300 µm fibre-optic handpiece, it was necessary to touch a special black paper so that all the light waves were directed into one point and reached their maximum efficiency before the beginning of the procedure. The optical tip of the handpiece was held angulated and away from the abutment teeth, moving circumferentially within the sulcus to remove the sulcular epithelium and give space for the impression material to be applied. Depending on the impression technique used, the sulcus needed to be thoroughly dried for the intraoral digital scanning, but not completely dry for the conventional impression material used due to its hydrophilicity. No local anaesthesia was administered prior to the gingival troughing procedure. Minor bleeding was controlled through the coagulative effect of the Nd: YAG laser and gentle air drying.

The 3M^TM^ Astringent Retraction Paste that was used for the gingival deflecting in the second group of teeth contains 15% aluminium chloride and comes in pre-dosed capsules that can be dispensed with the help of a composite dispenser. It was applied gently, steadily, inside the sulcus, keeping the tip of the capsule permanently directed towards the surface of the tooth, while moving it around the tooth from one proximal point on the buccal side to the same point on the lingual side, until the sulcus was filled with paste. After leaving the paste for 2 min, before taking the secondary impression, the sites where the paste was inserted were sprayed with an air-water spray to thoroughly wash away all the remaining paste before taking the secondary impression so that it would not influence the material’s setting process.

Before intraoral scanning, each sulcus was carefully rinsed and dried to standardize moisture conditions and ensure optimal scanning accuracy.

In the same visit, the secondary impressions were taken differently depending on the method used for tissue management. Because of the retraction paste’s time-sensitive properties, immediately after rinsing off the paste and thoroughly drying the field, a digital impression was taken for each dental arch. After reapplying and rinsing off the retraction paste, for each dental arch, the conventional impression was taken with polyether. After disinsertion, the impression was washed thoroughly under an abundant water jet and disinfected by immersion in 2% glutaraldehyde for 10 min, without affecting the polyether’s wettability.

Thus, 4 baseline and 4 secondary impressions were taken and sent physically and virtually to the dental laboratory, where the gypsum casts were poured for the conventional ones. The plaster casts were then scanned using a laboratory scanner: UP3D UP300E (UP3D Tech Co., Ltd., Shenzhen, China), which has an accuracy of 8 microns and can scan the entire arch in a few seconds. Following the scanning process, the files were saved in stereolithography format (STL file). Additionally, the digital impressions, with before and after gingival sulcus conditioning, were saved as STL files.

### 2.5. Data Analysis

The STL files were then analyzed using a specialized software designed for use in the dental laboratory: Exocad version 3.1 (Exocad GmbH, Darmstadt, Germany). In the ExoViewer3D program version 3.1 (Exocad GmbH, Darmstadt, Germany), two STL files for each dental arch and each type of impression were analyzed: four STL files containing the digital impressions taken before and after gingival displacement on each dental arch and another four STL files including the scanning of the dental casts obtained through conventional impression taking with polyether, summing a total of 8 such files.

The overlapping of the files was achieved using the “Best fit matching” function from the “Align Meshes” menu in the program. To evaluate the gingival displacement, the STL files obtained before and after the retraction procedure were imported into the Exocad software, version 3.1 (Exocad GmbH, Darmstadt, Germany). The two meshes were aligned using a point-based initial registration followed by a ‘Best-fit matching’ algorithm. This alignment was performed strictly on the non-deformable areas of the prepared teeth (the occlusal surface and the stable portions of the clinical crown) that remained unchanged during the retraction process. By using the ‘Best-fit’ alignment, a high degree of superimposition accuracy was achieved, ensuring that any visual discrepancy between the two models was strictly due to the horizontal displacement of the gingival margin and not to alignment errors. As a result of this procedure, a three-dimensional overlapped file was obtained, which was transferred to the DentalCAD program version 3.1 (Exocad GmbH, Darmstadt, Germany) to attain precise measurements and further conduct the analysis.

For each abutment tooth, three sections were then created, and measurements were taken at six distinct points, as listed below:

- Mesio-buccal (MB);
- Centro-buccal (CB);
- Disto-buccal (DB);
- Disto-oral (DO);
- Centro-oral (CO);
- Mesio-oral (MO).

The measurements at the six reference points were independently performed by two calibrated investigators who were not involved in the clinical procedures and who were blinded to the gingival displacement method used for each sample. The final value assigned to each measurement point was calculated as the mean of the two recorded values in order to minimize inter-observer variability.

**Figure 2:**
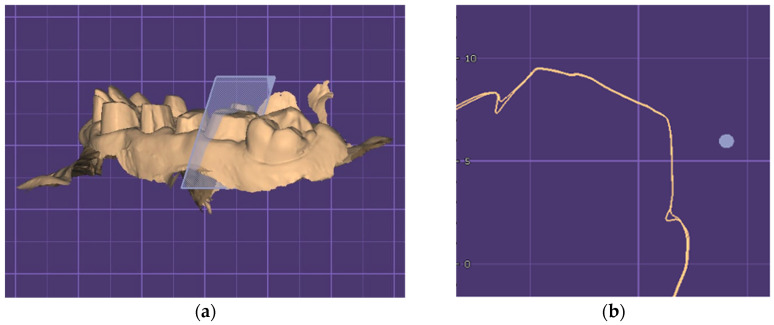
(**a**) Creating virtual sections for each abutment. (**b**) The sectional view that illustrates the coincidence of the two lines at the abutment level.

Figure 2a shows how the sections were virtually cut through the abutment and Figure 2b shows the proper overlapping of the meshes by tracking the coincidence of the lines that represent the contour of the abutment tooth, the free marginal gingiva, and its sulcus, one for each of the pre- and post-retraction phases. The only discrepancy between the two lines appears strictly at the level of the sulcus, where the displacement of the gingival tissue took place.

For each of the 6 surface points presented above, two measurements were taken on the sectional view of the overlapped meshes:

- 1st measurement—before the gingival displacement;
- 2nd measurement—after the gingival displacement.

**Figure 3:**
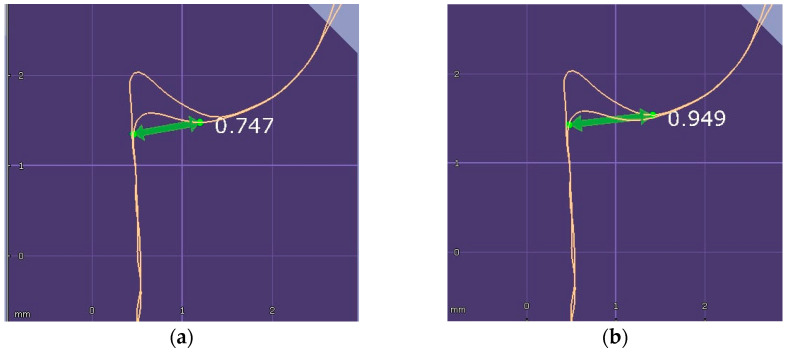
(**a**) The sulcus pre-retraction width. (**b**) The sulcus post-retraction width.

To measure the pre- and post-retraction width of the sulcus, a line was drawn perpendicularly from the most coronal point of the free marginal gingiva to the surface of the tooth. To ensure a stable and reproducible reference, the point on the tooth surface was selected precisely at the junction where the pre-displacement and post-displacement STL files coincided, as shown in Figure 3a,b.

The data were introduced in Excel (Microsoft, Redmond, WA, USA) tables with two values for each point: a pre-retraction value and a post-retraction one. The difference between them illustrates the horizontal expansion (sulcus width) at the gingival sulcus level. Afterward, a mean value was calculated for each abutment.

The primary endpoint of the study was defined as the mean sulcus width gain obtained after gingival displacement. Sulcus width gain was calculated as the difference between the pre-displacement and post-displacement measurements at each standardized reference point.

### 2.6. Statistical Analysis

Statistical analysis was performed using JASP software (version 0.18.3, University of Amsterdam, The Netherlands).

The intraclass correlation coefficient demonstrated a high level of agreement between the two examiners (ICC = 0.893; 95% CI: 0.841–0.928), indicating good inter-observer reliability of the digital measurements.

The normality of the data distribution was evaluated using the Shapiro–Wilk test. For normally distributed data, parametric tests (Welch’s *t*-test and paired *t*-test) were used, while the Mann–Whitney U test was applied for non-normally distributed datasets. The significance level (α) was set at 0.05.

Because multiple measurements were obtained from teeth belonging to the same patient, the measurements cannot be considered fully independent. Therefore, the statistical analysis should be interpreted with caution, as clustering effects may exist at the tooth and patient level. This limitation is inherent to the pilot design of the study.

## 3. Results

### 3.1. Sample Characteristics

This study compared the mean values of sulcus width gain after two different methods of tissue management: gingival troughing using Nd: YAG Laser and gingival displacement with Astringent Retraction Paste. The total number of teeth that were subjected to this study is represented by 114 sectional digital measurements, of which 60 were maxillary measurement points (52.63%), and 54 were mandibular (47.37%).

The mechano-chemical gingival displacement method using the Astringent Retraction Paste was applied on 57.89% of sections (66 measurement points), and the surgical gingival troughing method was used for 42.11% of teeth (48 measurement points).

All the teeth subjected to this study were scanned for both the baseline impression and the secondary impression with the intraoral scanning device (IOS). Also, the casts that were obtained after taking both the conventional baseline and secondary impressions were scanned with the dental laboratory scanning device to acquire the STL files. Regarding the distribution of the analyzed sections, 63.16% were located in the anterior (frontal) region, 31.58% in the premolar region, and 5.26% in the molar region, as shown in Table 1.

### 3.2. Comparative Results

#### 3.2.1. Intraoral Scanning (IOS)

Descriptive statistics for intraoral scanning measurements are presented in Table 2. The mean sulcus width gain following gingival troughing with the Nd: YAG laser was 0.270 ± 0.169 mm (95% CI: 0.223–0.317 mm), while the use of astringent retraction paste yielded a higher mean value of 0.320 ± 0.161 mm (95% CI: 0.281–0.359 mm). The coefficients of variation (0.626 and 0.505, respectively) suggest greater consistency for the paste group.

Normality testing using the Shapiro–Wilk test indicated that the data did not follow a normal distribution (*p* < 0.001). Therefore, a Mann–Whitney U test was applied to assess intergroup differences (Table 3), revealing a non-significant trend towards higher gingival displacement values in the paste group (*p* = 0.064).

**Figure 4:**
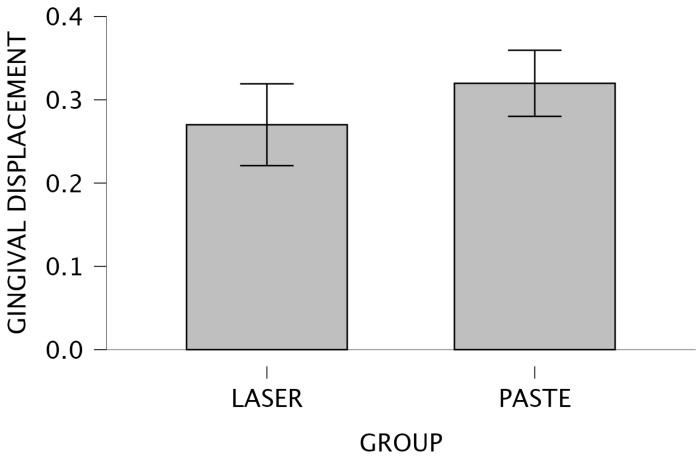
Bar plot—Intergroup comparison for IOS.

**Figure 5:**
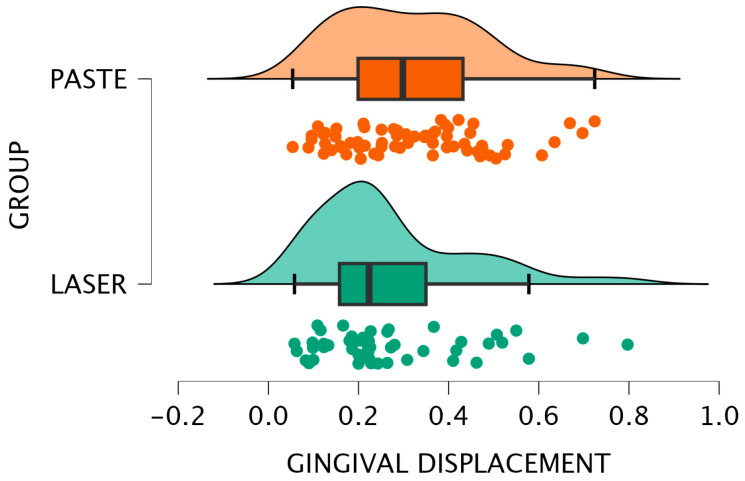
Raincloud plot—Intergroup comparison for IOS.

Figure 4 and Figure 5 illustrate this comparison through a bar plot and a raincloud plot, respectively, highlighting the slightly higher central tendency in the paste group.

#### 3.2.2. Laboratory Scanning Method (LABSCAN)

For the laboratory scanner, descriptive data are summarized in Table 4. The mean gain in sulcus width was 0.333 ± 0.117 mm (95% CI: 0.300—0.366 mm) for the Nd: YAG laser and 0.378 ± 0.171 mm (95% CI: 0.337—0.419 mm) for the retraction paste. The Shapiro–Wilk test confirmed normal data distribution (*p* = 0.118), allowing the use of Welch’s *t*-test for intergroup comparison.

The test (Table 5) revealed no statistically significant difference between the two techniques (*p* = 0.099), although higher mean values were again observed for the astringent paste group.

**Figure 6:**
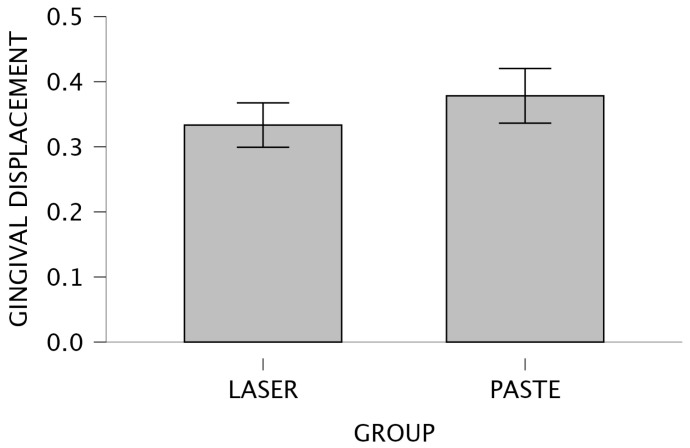
Bar plot—Intergroup comparison for LABSCAN.

**Figure 7:**
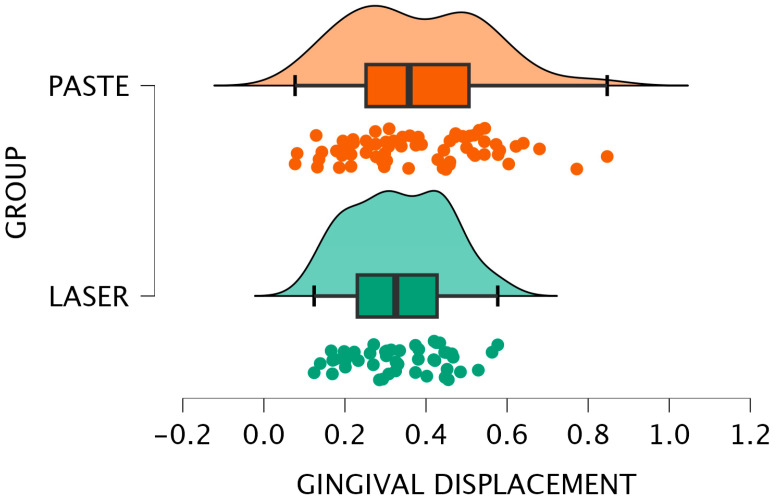
Raincloud plot–Intergroup comparison for LABSCAN.

Figure 6 and Figure 7 display the results graphically, confirming a consistent pattern of slightly increased displacement with the paste method, regardless of the scanning approach used.

#### 3.2.3. Differences Between Scanning Methods

When comparing the two scanning modalities (Table 6), the mean displacement values were 0.299 ± 0.166 mm (95% CI: 0.268–0.330 mm) for the intraoral scanner and 0.359 ± 0.152 mm (95% CI: 0.332–0.386 mm) for the laboratory scanner. Normality testing showed no significant deviations (*p* = 0.401), allowing a paired *t*-test to be performed.

The results (Table 7) demonstrated a statistically significant difference between the two scanning systems (*p* < 0.001).

**Figure 8:**
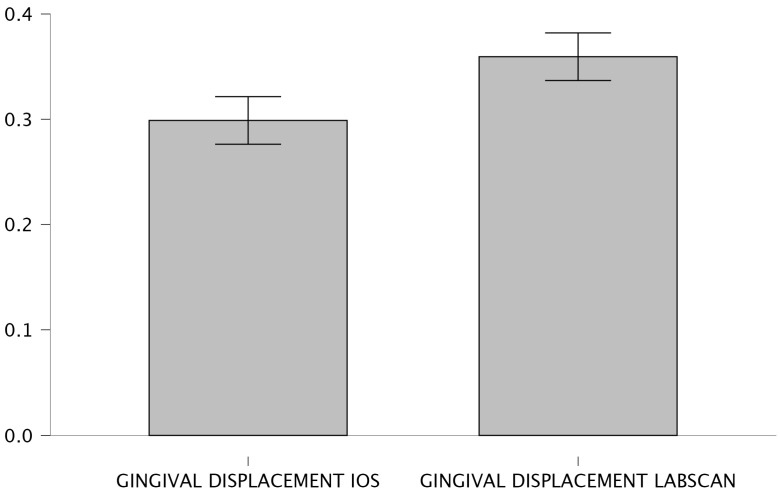
Bar plot—Comparison between scanners.

**Figure 9:**
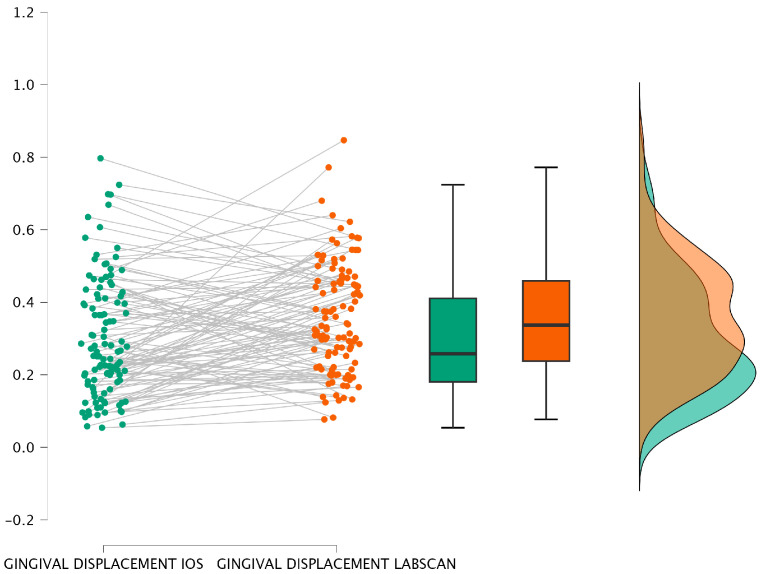
Raincloud plot- Comparison between scanners.

The corresponding graphical representations (Figure 8 and Figure 9) show a clear visual separation of the mean values, confirming that measurements obtained with the laboratory scanner tend to indicate a greater horizontal displacement of the gingival sulcus.

## 4. Discussion

Every dental clinician aims to achieve satisfactory results in oral rehabilitation. Thus, it is necessary to attain a highly accurate impression. To accomplish this, the gingival sulcus goes through a process of both horizontal and vertical expansion. According to the literature, an impression without imperfections has been observed after minimal deflection of the gingival sulcus of 0.20 mm or 0.22 mm [3,11]. The existence of numerous impression materials on the market and different gingival troughing techniques has made achieving this aim possible today. Both investigated methods exceeded the minimum gingival displacement threshold of 0.20 mm required to obtain an accurate impression. The mean sulcus width gain ranged from 0.270 mm for the Nd: YAG laser using intraoral scanning to 0.378 mm for the astringent retraction paste evaluated with the laboratory scanner. Although all values met the minimal criterion, higher mean values were consistently observed for the astringent paste compared to the laser method and the laboratory scanner compared to the intraoral scanner.

The present study should primarily be interpreted as a methodological pilot investigation exploring the feasibility of digital STL analysis for evaluating gingival sulcus width changes.

Given that the existing literature contains a substantial number of studies evaluating gingival retraction cords, both impregnated and non-impregnated, their clinical effectiveness and limitations are already well documented. In this context, the present study deliberately focused on the assessment of two more recently introduced techniques for gingival sulcus expansion. By investigating Nd: YAG laser troughing and an astringent retraction paste, our aim was to explore alternative soft tissue management approaches that align with contemporary digital workflows and may offer practical advantages over conventional cord-based methods. Merve Benli reported that astringent retraction cords produced the greatest horizontal gingival displacement (0.72 ± 0.08 mm), values that are considerably higher than those obtained in the present study. In the same investigation, the use of Expasyl paste (Acteon Group, Mérignac, France) resulted in lower displacement values (0.48 ± 0.03 mm). However, these values still exceeded the horizontal gingival displacement achieved with the astringent paste evaluated in our study [12].

In the study conducted by Singh Amith A. et al., the effectiveness of different gingival retraction methods was evaluated in terms of horizontal gingival displacement. The authors reported a mean horizontal displacement of 0.767 mm when using plain retraction cord, while a comparable mean displacement of 0.757 mm was achieved with the magic foam cord. The minimal difference observed between the two techniques suggests that both methods provide a similar capacity for lateral gingival displacement. These findings indicate that alternative, less invasive retraction systems such as a magic foam cord system may offer clinical effectiveness comparable to conventional retraction cords, potentially with improved patient comfort and reduced tissue trauma, although further studies are required to confirm these advantages under different clinical conditions [13].

Polyether, Impregum^TM^ (ESPE GmbH, Germany) was one of the first polyethers that appeared on the market. This impression material is hydrophilic, so it can be used in humid conditions, similar to the oral cavity. In addition, this specific characteristic allows the dental gypsum to flow more easily in every detail of the impression, resulting in a very precise cast [14]. As a consequence of the material’s water-absorbing characteristic, the impression should not be submerged in water or any other substances for decontamination purposes for an extended period, as this may result in dimensional changes of the impression [15,16,17].

In recent times, the advancement of new technologies has significantly impacted the fields of biomedicine, medicine, and biotechnology, leading to improvements that benefit both clinicians and patients. A notable innovation in modern dental impressions involves the introduction of digital scanning devices, which are capable of capturing a large number of images of dental arches and subsequently processing them using specialized software [18].

One of the most notable advantages of utilizing such an advanced technology as the intraoral scanner is the substantial reduction in discomfort experienced by patients, who often feel apprehensive about undergoing traditional impression methods involving impression trays and materials like alginates, silicones, or polyethers. Intraoral scanning technology is very versatile and can be applied to various procedures, including the fabrication of both fixed and removable dental prostheses, orthodontic treatments, and the detection of tooth decay. However, the main drawback of intraoral scanners is their significantly high purchase cost. Additionally, healthcare professionals and operators must acquire and master complex skills to utilize the device [18].

Gingival displacement with astringent retraction paste and gingival troughing with a Nd: YAG laser are procedures that allow the impression material to flow deeper into the gingival sulcus [19]. Also, both methods modulate the moisture in the gingival sulcus, creating the necessary conditions for digital impression. The Nd: YAG laser brings a new concept in dental treatments with increased efficiency in disinfection and coagulation, while also being used in gingival eviction [20]. The effectiveness of gingival width gain with laser systems can be noticed with a gingival troughing of 0.230–0.670 mm, wider than the minimum level mentioned above [6,21,22]. In our study, a mean value of gingival troughing of 0.270 mm for the IOS and 0.330 mm for the method using the laboratory scanner was achieved. With similar settings used for gingival eviction, Xian Tao et al. obtained values that exceeded the minimum limit for obtaining a void-free impression [23]. These preliminary preparations are necessary to obtain a clean and dry area in the absence of saliva and blood [24,25].

Xian Tao et al. reported values of 0.60 ± 0.17 mm for Nd: YAG gingival troughing [23], compared to our 0.270 mm for IOS and 0.333 mm for LABSCAN. The differences between the two gingival displacement methods in our study were not statistically significant. In this study, the lower mean values obtained in the Nd: YAG laser group were observed despite the use of a 300 µm optical fiber, which would theoretically be expected to produce a broader ablation zone and enhanced lateral gingival tissue removal, thereby facilitating greater sulcus widening. According to the literature, fiber diameter plays a significant role in energy delivery and tissue interaction. Therefore, the lower-than-expected values obtained in the present investigation suggest that additional factors, such as laser settings, tissue characteristics, or operator-dependent variables, may have influenced the extent of gingival displacement. Hence, although our laser protocol achieved clinically acceptable results, future investigations might benefit from standardizing fiber diameter and optimizing delivery parameters to potentially improve gingival troughing performance.

The use of the astringent retraction paste is much easier compared to the retraction cord. The pre-dosed capsule system presents several advantages: the capsule’s fine tip (that can easily be inserted into the sulcus), hygienic application (single-use capsules), and ease of application with the composite dispensers [26]. The astringent paste must be rinsed very well after the retraction time because it may interfere with the setting process of the impression material. One study showed that the astringent retraction paste produced the highest amount of gingival displacement with a mean value of 500 μm [27,28], a higher value compared to our results (0.32 mm for intraoral scanning and 0.378 mm for laboratory cast scanning). Even if we observed a higher value of gingival sulcus width gain after the use of astringent retraction paste compared to the gingival troughing, the difference was not statistically significant, as proved after the application of the statistical tests.

Higher values were observed for the method that used the laboratory scanner, probably due to the use of a polyether impression material, and ll the tooth surfaces were imprinted at the same time. Another cause could be the higher accuracy of the UP3D scanner compared to the Medit i500 scanner. Each of the scanning methods has advantages and disadvantages. According to a study published in 2021, obtaining the STL files by intraoral scanning showed the highest trueness, while obtaining them with a laboratory scanner showed the highest precision [29]. The marginal fit of fixed restorations showed higher accuracy for the digital method than the conventional one [30], but full arch scans are more challenging and technique-sensitive [27]. Previous studies have reported that laboratory scanners may demonstrate higher precision in controlled conditions. However, the present study did not evaluate scanner precision directly but only compared the sulcus width measurements obtained using two acquisition methods.

An in vitro evaluation of the trueness of dental impressions obtained using digital scanners compared the UP3D scanner with three other devices, including the MEDIT system, and reported that the UP3D scanner, which operates on structured light technology, exhibited high trueness among the evaluated scanners. However, a notable limitation of that study is the lack of detailed information regarding the specific models and technical characteristics of the scanners used, which restricts the comparability of the results [31]. In the present study, a statistically significant difference was observed between the two scanning techniques, with higher mean values recorded for the laboratory scanner compared to the intraoral scanner (*p* < 0.001). The observed differences between intraoral and laboratory scanning measurements may be influenced by several technical factors, including scanner resolution, mesh reconstruction algorithms, and the dimensional behavior of impression materials used for cast fabrication. This finding suggests that the method of data acquisition influences the measurement outcome, as laboratory scanning tends to capture slightly larger sulcus width values, likely due to the combined effects of impression material behavior and scanning precision.

The present study was designed as a preliminary investigation focusing on feasibility and methodological validation rather than definitive hypothesis testing. Consequently, the reduced sample size represents an inherent limitation of the study and may have limited the ability to detect small intergroup differences. Although the intergroup differences were not statistically significant for either intraoral or laboratory scanning, a significant difference was observed between the two scanning techniques, in favor of the laboratory scanner. These findings indicate that the method of digital data acquisition may influence the measured outcomes, emphasizing the role of scanning precision and impression material behavior in reproducing fine gingival details.

Our results should be interpreted with caution, given the limited sample size and the exploratory nature of the study. The limitations of this preliminary work mainly include the small sample size, the sensitivity of intraoral scanners to oral fluids, and the technique-dependent challenges of digital impression procedures. Furthermore, the multiple steps required to obtain STL files via laboratory scanning introduce potential errors at several stages of data acquisition.

Additional factors that may have influenced the results include the variation in gingival biotypes (thin vs. thick), which can affect the tissue response to gingival displacement procedures, as well as the non-uniform distribution of tooth regions within the sample. The gingival tissue presents distinct morphological and biomechanical characteristics in anterior, premolar, and molar areas, potentially leading to slight discrepancies in sulcus width gain among different regions of the arch. Moreover, the study did not assess the healing dynamics of the gingival tissues, which is a relevant parameter, since the use of lasers, retraction cords, or astringent pastes can induce recession or marginal remodeling over time. The short interval between tooth preparation and gingival displacement procedures may also have influenced soft tissue stability. In addition, the biologically oriented preparation technique (BOPT) employed in this study might have affected the gingival margin behavior, further influencing the outcomes. Additionally, this type of preparation may represent a source of bias about the accuracy and reproducibility of the measurements. In most previously published studies, gingival displacement is measured relative to a clearly defined preparation margin, which provides a stable and reproducible reference point [32,33,34]. In contrast, the absence of a distinct finish line in BOPT required the use of an empirically selected reference point on the tooth surface, which may have influenced measurement precision. Future studies should consider the use of a chamfer or shoulder preparation with a clearly defined margin in order to improve measurement reproducibility and enhance the validity of comparative analyses.

Future studies with larger and more homogeneous samples, including standardized distribution across dental regions and longer follow-up periods, are necessary to validate these findings. Expanding this line of research will contribute to developing evidence-based clinical guidelines for optimal soft tissue management and impression accuracy in modern prosthodontic workflows.

## 5. Conclusions

Although intergroup differences were not statistically significant, a significant variation was observed between the scanning modalities, with higher sulcus width measurements recorded for the laboratory scanner.

These findings highlight the influence of the scanning technique on measurement outcomes and suggest that both gingival displacement approaches may be clinically viable. Further clinical studies with larger samples and standardized protocols are required to confirm these preliminary findings.

## Figures and Tables

**Table 1:** Distribution of measurement points according to tooth region.

Tooth Region	Number of Measurements (*n*)	Percentage (%)
Frontal	72	63.16%
Premolar	36	31.58%
Molar	6	5.26%
Total	114	100%

**Table 2:** Group Descriptives for IOS.

	Group	*n*	Mean	SD	SE	Coefficient of Variation	Mean Rank	Sum Rank	95% CI
Gingival displacement	LASER	48	0.27	0.169	0.024	0.626	50.77	2437	0.223–0.317
	PASTE	66	0.32	0.161	0.02	0.505	62.39	4118	0.281–0.359

SD: standard deviation; SE: standard error; CI: confidence interval.

**Table 3:** Mann-Whitney U test.

	U	df	*p*
Gingival displacement	1261		0.064

**Table 4:** Group Descriptives for LABSCAN.

-	Group	*n*	Mean	SD	SE	Coefficient of Variation	95% CI
Gingival displacement	Laser	48	0.333	0.117	0.017	0.352	0.300–0.366 mm
	Paste	66	0.378	0.171	0.021	0.451	0.337–0.419 mm

**Table 5:** Welch’s *t*-test.

	t	df	*p*	Mean Difference	SE Difference
Gingival displacement	−1.663	111.7	0.099	−0.045	0.027

**Table 6:** Descriptive Statistics for both scanning methods.

	*n*	Mean	SD	SE	Coefficient of Variation	95% CI
Gingival displacement IOS	114	0.299	0.166	0.016	0.555	0.268–0.330
Gingival displacement LABSCAN	114	0.359	0.152	0.014	0.422	0.332–0.386

**Table 7:** Paired Samples *t*-test.

Measure 1	Measure 2	t	df	*p*
Gingival displacement IOS	Gingival displacement LABSCAN	−3.752	113	<0.001

## Data Availability

Data is contained within the article.

## Abbreviations

The following abbreviations are used in this manuscript:

Nd: YAG	Neodymium-doped Yttrium Aluminum Garnet
STL	Stereolithography
IOS	Intraoral Scanner
LABSCAN	Laboratory Scanner
PPD	Probing Pocket Depth
GR	Gingival Retraction
PI	Plaque Index
BOP	Bleeding on Probing
BOPT	Biologically Oriented Preparation Technique
OPG	Orthopantomography
JASP	Jeffrey’s Amazing Statistics Program
SD	Standard Deviation
SE	Standard Error
df	Degrees of Freedom
α	Significance Level

## References

[1] Frățilă A., Crișan T. (2022). Subgingival Localization of the Prosthetic Restorations and Periodontal Health. Acta Medica Transilv..

[2] Baba N.Z., Goodacre C.J., Jekki R., Won J. (2014). Gingival Displacement for Impression Making in Fixed Prosthodontics: Contemporary Principles, Materials, and Techniques. Dent. Clin. N. Am..

[3] Gajbhiye V., Banerjee R., Jaiswal P., Chandak A., Radke U. (2019). Comparative Evaluation of Three Gingival Displacement Materials for Efficacy in Tissue Management and Dimensional Accuracy. J. Indian Prosthodont. Soc..

[4] Baharav H., Laufer B.Z., Langer Y., Cardash H.S. (1997). The Effect of Displacement Time on Gingival Crevice Width. Int. J. Prosthodont..

[5] Safari S., Ma V.S., Mi V.S., Hamed M. (2016). Gingival Retraction Methods for Fabrication of Fixed Partial Denture: Literature Review. J. Dent. Biomater..

[6] Sorrentino R., Ruggiero G., Zarone F. (2022). Laser Systems for Gingival Retraction in Fixed Prosthodontics: A Narrative Review. J. Osseointegration.

[7] Indriyani A., Masulili C., Odang R.W.L. (2020). Effect of Gingival Retraction Method to Lateral Gingival Displacement Width. Pesqui. Bras. Odontopediatria Clín. Integr..

[8] Lim J.-H., Mangal U., Nam N.-E., Choi S.-H., Shim J.-S., Kim J.-E. (2021). A Comparison of Accuracy of Different Dental Restorative Materials between Intraoral Scanning and Conventional Impression-Taking: An In Vitro Study. Materials.

[9] Berrendero S., Salido M.P., Ferreiroa A., Valverde A., Pradíes G. (2019). Comparative Study of All-Ceramic Crowns Obtained from Conventional and Digital Impressions: Clinical Findings. Clin. Oral Investig..

[10] Gupta R., Brizuela M. Dental Impression Materials. http://www.ncbi.nlm.nih.gov/books/NBK574496/.

[11] Yilmaz H., Aydin C., Gul B., Yilmaz C., Semiz M. (2007). Effect of Disinfection on the Dimensional Stability of Polyether Impression Materials. J. Prosthodont..

[12] Khatri M., Mantri S.S., Deogade S.C., Bhasin A., Mantri S., Khatri N., Jain P., Chauhan D. (2020). Effect of Chemical Disinfection on Surface Detail Reproduction and Dimensional Stability of a New Vinyl Polyether Silicone Elastomeric Impression Material. Contemp. Clin. Dent..

[13] Apinsathanon P., Bhattarai B.P., Suphangul S., Wongsirichat N., Aimjirakul N. (2022). Penetration and Tensile Strength of Various Impression Materials of Vinylsiloxanether, Polyether, and Polyvinylsiloxane Impression Materials. Eur. J. Dent..

[14] Cicciù M., Fiorillo L., D’Amico C., Gambino D., Amantia E.M., Laino L., Crimi S., Campagna P., Bianchi A., Herford A.S. (2020). 3D Digital Impression Systems Compared with Traditional Techniques in Dentistry: A Recent Data Systematic Review. Materials.

[15] Kuhn K., Zügel D., Korbay V.-S.A., Papas T., Schnutenhaus S., Luthardt R.G., Dreyhaupt J., Rudolph H. (2022). Gingival Displacement in the Vertical and Horizontal Dimension under the Condition of Mild Gingivitis—A Randomized Clinical Study. J. Clin. Med..

[16] Maden I., Maden O.E., Kazak Z. (2013). The TwinLightTM Concept in Dentistry. LAHA.

[17] Wassell R., Nohl F., Steele J., Walls A. (2019). Extra-Coronal Restorations: Concepts and Clinical Application.

[18] Krishna Ch V., Gupta N., Reddy K.M., Sekhar N.C., Aditya V., Reddy G.V.K.M. (2013). Laser Gingival Retraction: A Quantitative Assessment. J. Clin. Diagn. Res. JCDR.

[19] Tao X., Yao J.-W., Wang H.-L., Huang C. (2018). Comparison of Gingival Troughing by Laser and Retraction Cord. Int. J. Periodontics Restor. Dent..

[20] Ünalan Değirmenci B., Karadağ Naldemir B., Değirmenci A. (2021). Evaluation of Gingival Displacement Methods in Terms of Periodontal Health at Crown Restorations Produced by Digital Scan: 1-Year Clinical Follow-Up. Lasers Med. Sci..

[21] Goutham G.B., Jayanti I., Jalaluddin M., Avijeeta A., Ramanna P.K., Joy J. (2018). Clinical Assessment of Gingival Sulcus Width Using Various Gingival Displacement Materials. J. Contemp. Dent. Pract..

[22] Burke F.T., Crisp R.J. (2014). Evaluation of a Novel Compule-Based Gingival Retraction System in UK General Dental Practices. Dent. Update.

[23] Qureshi S.M., Anasane N.S., Kakade D. (2020). Comparative Evaluation of the Amount of Gingival Displacement Using Three Recent Gingival Retraction Systems—In Vivo Study. Contemp. Clin. Dent..

[24] 3M ESPE Retraction Capsule—The Dental Advisor. https://www.dentaladvisor.com/evaluations/3m-espe-retraction-capsule/.

[25] Kontis P., Güth J.-F., Schubert O., Keul C. (2021). Accuracy of Intraoral Scans of Edentulous Jaws with Different Generations of Intraoral Scanners Compared to Laboratory Scans. J. Adv. Prosthodont..

[26] Rech-Ortega C., Fernandez-Estevan L., Sola-Ruiz M., Agustin-Panadero R., Labaig-Rueda C. (2018). Comparative in Vitro Study of the Accuracy of Impression Techniques for Dental Implants: Direct Technique with an Elastomeric Impression Material versus Intraoral Scanner. Med. Oral Patol. Oral Cir. Bucal.

[27] Nulty A.B. (2021). A Comparison of Full Arch Trueness and Precision of Nine Intra-Oral Digital Scanners and Four Lab Digital Scanners. Dent. J..

[28] Sindhu S., Maiti S., Nallaswamy D. (2023). Factors Affecting the Accuracy of Intraoral Scanners-A Systematic Review. Ann. Dent. Spec..

[29] Rapone B., Palmisano C., Ferrara E., Di Venere D., Albanese G., Corsalini M. (2020). The Accuracy of Three Intraoral Scanners in the Oral Environment with and without Saliva: A Comparative Study. Appl. Sci..

[30] Agustín-Panadero R., Moreno D.M., Pérez-Barquero J.A., Fernández-Estevan L., Gómez-Polo M., Revilla-León M. (2023). Influence of Type of Restorative Materials and Surface Wetness Conditions on Intraoral Scanning Accuracy. J. Dent..

[31] Bindhu Sree C., Koka P., Sri I.K., Alex J.A., Rani K.D., Chander V.B. (2025). Comparative Evaluation of Trueness of Dental Impressions Using Digital Scanners: An In Vitro Study. J. Pharm. Bioallied Sci..

[32] El Ashry M.F., Abdelkader S.H., Hammad I.A., Fahmy R.A., Abdelraheem I.M. (2025). The Efficacy of Different Gingival Displacement Methods for Definitive Digital Impressions: A Randomized Controlled Trial. J. Dent..

[33] Kumar A., Nandini V.V., Boruah S., Jailance L. (2024). Comparison of Gingival Displacement Using Paste Technique and Combination Technique (Cord and Paste) in Digital Impressions: A Pilot Study. J. Orofac. Rehabil..

[34] Jain M., Taneja S., Katyal S. (2025). Comparative Evaluation of Gingival Displacement and Inflammatory Response Using Different Retraction Techniques—A Randomized Controlled Trial. J. Conserv. Dent. Endod..

